# Correction of serum potassium with sodium zirconium cyclosilicate in Japanese patients with hyperkalemia: a randomized, dose–response, phase 2/3 study

**DOI:** 10.1007/s10157-020-01937-1

**Published:** 2020-08-10

**Authors:** Naoki Kashihara, Toshiki Nishio, Takeshi Osonoi, Yosuke Saka, Toshiyuki Imasawa, Takayasu Ohtake, Hiroshi Mizuno, Yugo Shibagaki, Hyosung Kim, Toshitaka Yajima, Nobuaki Sarai

**Affiliations:** 1 Department of Nephrology and Hypertension, Kawasaki Medical School, Okayama, Japan; 2 Nephrology Dialysis Center, Kusatsu General Hospital, Shiga, Japan; 3 Department of Internal Medicine, Nakakinen Clinic, Ibaraki, Japan; 4 Department of Nephrology, Kasugai Municipal Hospital, Aichi, Japan; 5 Department of Nephrology, Chiba-Higashi Hospital, Chiba, Japan; 6 Department of Nephrology, Shonan Kamakura General Hospital, Kanagawa, Japan; 7 Department of Nephrology, Inage Hospital, Chiba, Japan; 8 Division of Nephrology and Hypertension, St. Marianna University School of Medicine Hospital, Kanagawa, Japan; 9 Research and Development, AstraZeneca K.K, 1-8-3, Marunouchi, Chiyoda-ku, Tokyo, 100-0005 Japan

**Keywords:** Sodium zirconium cyclosilicate, Hyperkalemia, Japanese, Japan

## Abstract

**Background:**

Sodium zirconium cyclosilicate (SZC) is an oral potassium binder approved to treat hyperkalemia in adults in a number of countries, including Japan.

**Methods:**

This phase 2/3, randomized, double-blind, placebo-controlled, dose–response study (ClinicalTrials.gov: NCT03127644) was designed to determine the efficacy and safety of SZC in Japanese adults with hyperkalemia. Patients with serum potassium (sK^+^) concentrations ≥ 5.1– ≤ 6.5 mmol/L were randomized 1:1:1 to SZC 5 g, SZC 10 g, or placebo three times daily for 48 h (six doses total). The primary efficacy endpoint was the exponential rate of change in sK^+^ over 48 h. The proportion of patients with normokalemia (sK^+^ 3.5–5.0 mmol/L) at 48 h and adverse events (AEs) were also evaluated.

**Results:**

Overall, 103 patients (mean age, 73.2 years; range 50–89 years) received SZC 5 g (*n* = 34), SZC 10 g (*n* = 36), or placebo (*n* = 33). The exponential rate of sK^+^ change from 0 to 48 h versus placebo was − 0.00261 (SZC 5 g) and – 0.00496 (SZC 10 g; both *P* < 0.0001). At 48 h, the proportions of patients with normokalemia were 85.3%, 91.7%, and 15.2% with SZC 5 g, SZC 10 g, and placebo, respectively. No serious AEs were reported. Hypokalemia (sK^+^  < 3.5 mmol/L) occurred in two patients in the SZC 10 g group; normokalemia was re-established within 6 days and no treatment-related AEs were reported.

**Conclusion:**

SZC is effective and well tolerated in Japanese patients with hyperkalemia.

**Electronic supplementary material:**

The online version of this article (10.1007/s10157-020-01937-1) contains supplementary material, which is available to authorized users.

## Introduction

Hyperkalemia, a common electrolyte disorder defined by elevated serum potassium (sK^+^), is often associated with comorbidities such as chronic kidney disease (CKD), heart failure (HF), and diabetes [1]. Hyperkalemia is also a common side effect of renin–angiotensin–aldosterone system inhibitors (RAASi), used in the management of CKD and HF [2–4]. In Japan, the overall prevalence of hyperkalemia in a hospital claims database population was 67.9 patients per 1000; this increased to 227.9, 134.0, 108.4, and 142.2 patients per 1000 in those with CKD, HF, diabetes, and RAASi therapy, respectively [1].

Severe hyperkalemia can cause serious cardiac arrhythmia [5], and is associated with increased all-cause, in-hospital, and cardiovascular death [6–8]. Large observational studies showed a U-shaped relationship between sK^+^ and mortality [9, 10], with the 3-year incidence of in-hospital death in Japanese patients lowest with sK^+^ 4.0 mmol/L, and the risk of death increasing   over seven-fold at concentrations of 5.1–5.4 mmol/L [1].

Options for managing hyperkalemia in Japan are limited, relying on dietary restrictions, cessation or modification of inciting agents, and potassium elimination with non-specific exchange resins, e.g., sodium polystyrene sulfonate (SPS) and calcium polystyrene sulfonate (CPS) [11, 12]. Although SPS and CPS have been widely used for several decades, their efficacy and safety have been questioned [13–16]. There is a significant need for novel treatments that correct and maintain sK^+^ within a safe range in Japanese patients with hyperkalemia.

Sodium zirconium cyclosilicate (SZC) is a novel, non-absorbed, highly selective potassium binder which preferentially entraps potassium ions in the gastrointestinal lumen in exchange for hydrogen and sodium ions [17–19]. Binding of free potassium in the gastrointestinal lumen by SZC increases fecal potassium excretion, consequently lowering sK^+^ concentration [17, 19, 20]. The efficacy and safety of SZC were demonstrated in phase 3 studies of patients with hyperkalemia with and without CKD or HF [18, 20, 21]. In these studies, SZC 10 g three times daily (TID) reduced mean sK^+^ concentration to within the normal range within 48 h, significantly reducing sK^+^ versus placebo as early as 1-h post-baseline [18, 20]. Sustained potassium control for up to 1 year has been demonstrated with SZC [18, 20–22]. SZC is currently approved in a number of countries, including Japan, for the treatment of hyperkalemia in adults [23–25].

Phase 3 studies of SZC performed to date included relatively few patients of Asian ethnicity [18, 20, 21]. However, potassium intake varies between different countries and cultures [26]; notably, consumption of potassium in the Japanese population is lower than that of Western countries [27]. Therefore, we performed a phase 2/3 dose–response study to determine the efficacy and safety of SZC in Japanese patients with hyperkalemia.

## Methods

### Study design

This phase 2/3, multicenter, randomized, double-blind, placebo-controlled, dose–response confirmatory study (ClinicalTrials.gov: NCT03127644) was conducted at 24 investigational sites in Japan between June 14, 2017 and February 23, 2018. See Supplemental Materials for ethical permissions and patient consent procedures.

The study comprised a screening visit, a 48-h treatment period, and a 1-week follow-up visit (± 2 days) (Fig. 1). In the treatment period, patients were randomized 1:1:1 to receive double-blind treatment with SZC (5 g or 10 g) or placebo TID for 48 h (six doses total); see Supplemental Materials for details.

**Fig. 1:**
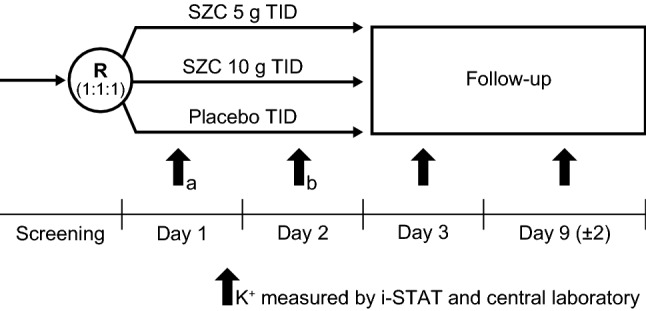
Study design. ^a^Potassium concentrations measured before, and 1, 2, and 4 h after administration of the first dose. ^b^Potassium concentrations measured before, and 1 and 4 h after the first dose. Randomization was stratified according to estimated glomerular filtration rate < 60 mL/min/1.73 m^2^ or ≥ 60 mL/min/1.73 m^2^. *K*^+^ potassium, *R* randomization, *SZC* sodium zirconium cyclosilicate, *TID* three times daily

### Patients

Eligible patients were adults aged ≥ 18 years with hyperkalemia, defined as two consecutive potassium concentrations of ≥ 5.1 to ≤ 6.5 mmol/L, measured 60 (± 10) min apart and within 1 day before study drug administration, using a point-of-care i-STAT device (Abbott Point of Care, Inc, Princeton, New Jersey, US). Key exclusion criteria were symptoms or causes of pseudohyperkalemia, diabetic ketoacidosis, cardiac arrhythmia requiring immediate treatment, dialysis, and treatment with non-absorbed antibiotics for hyperammonemia, resins, calcium acetate, calcium carbonate, or lanthanum carbonate within 7 days before the first dose of study drug. See Supplemental Materials for full eligibility criteria.

Patients discontinued the study if they developed serious cardiac arrhythmia, acute HF, or potential hyperkalemia-related electrocardiogram (ECG) changes, or if their i-STAT potassium concentration was > 6.5 mmol/L. Patients discontinued study treatment if they had a confirmed i-STAT potassium concentration of < 3.0 mmol/L. Patients with i-STAT potassium levels ≥ 3.0 to ≤ 3.4 mmol/L on days 1 or 2 were to discontinue the study drug for the remainder of the day and return the next day for evaluation to determine whether treatment could be continued.

### Assessments

All potassium samples were measured using both an i-STAT point-of-care device and by the central laboratory. Real-time assessment of potassium by i-STAT was used to determine study eligibility and to guide treatment decisions. Efficacy was evaluated using central laboratory measurements of sK^+^. Potassium concentrations were measured prior to study drug administration. Patients attended the clinic on day 1 (visit 2), day 2 (visit 3), day 3 (visit 4), and follow-up period (end of study), and fasted for a minimum of 8 h prior to blood sampling. Safety assessments included AE recording, physical examination, vital signs, laboratory measures, and ECG. Study assessments are detailed in the Supplemental Materials.

During the study, patients could not receive alternative hyperkalemia treatment (including medication). No dietary recommendations or restrictions to limit potassium intake were advised to patients. Insulin use was not restricted; all serum measurements were collected prior to insulin treatment. Oral medications with gastric pH-dependent bioavailability were administered at least 2 h before or after study drug to mitigate the risk of drug–drug interactions (Supplemental Materials).

### Study endpoints

The primary efficacy endpoint was the exponential rate of change in sK^+^ concentration during the initial 48 h of treatment. Baseline sK^+^ measurements are summarized in the Supplemental Materials.

The key secondary efficacy endpoint was the proportion of patients achieving normokalemia (sK^+^ 3.5–5.0 mmol/L) at 48 h after first dose. Other secondary efficacy endpoints included the proportion of patients achieving normokalemia at 48 h and at each scheduled sK^+^ assessment time point after start of dosing, mean change from baseline in sK^+^ concentration at all time points post-dose, and time to normalization of sK^+^ concentration.

Safety, tolerability, and treatment compliance were also assessed (Supplemental Materials).

### Statistical analysis

All statistical analyses were performed using SAS® version 9.4 (SAS Institute, Inc, Cary, North Carolina, US). Target sample size calculations are described in the Supplemental Materials.

Efficacy analyses were performed on all randomized patients (full analysis set). Exponential rate of change was analyzed using a random coefficient model. Estimates of treatment difference in slopes (i.e., coefficients for the time by treatment interaction for each dose) are presented for both SZC groups with 95% CIs as well as two-sided *P* values for pairwise comparison with placebo. Further details of the statistical methods for efficacy evaluation, including sensitivity and subgroup analyses are summarized in the Supplemental Materials.

Safety analyses were performed in all patients receiving ≥ 1 dose of study drug (safety analysis set) summarized in the Supplemental Materials.

## Results

### Patient disposition and baseline characteristics

Of 151 patients enrolled, 48 did not meet eligibility criteria; thus, 103 patients were randomized (SZC 5 g, *n* = 34; SZC 10 g, *n* = 36; placebo, *n* = 33; Fig. 2). The study was completed by 101 patients (98.1%); two patients (1.9%) in the placebo group discontinued after developing sK^+^ concentrations > 6.5 mmol/L.

**Fig. 2:**
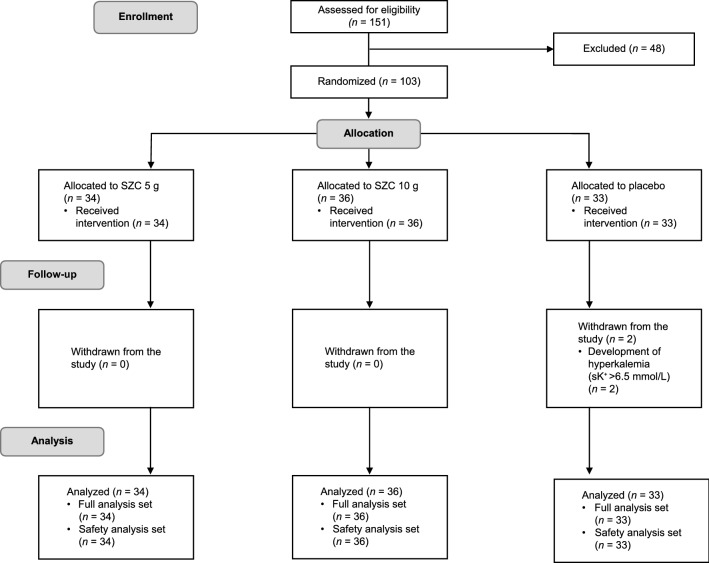
Study flow diagram. Randomization was stratified according to estimated glomerular filtration rate < 60 mL/min/1.73 m^2^ or ≥ 60 mL/min/1.73 m^2^. *sK*^+^ serum potassium, *SZC* sodium zirconium cyclosilicate

Baseline patient characteristics were generally well balanced across treatment groups (Table 1). Mean age was 73.2 years (range 50–89 years) and 74.8% were male. Most patients had comorbidities based on medical history, and all patients had received prior and concomitant medications (Table 1).

**Table 1:** Patient demographics and baseline characteristics (full analysis set)

Characteristic	SZC 5 g (*n* = 34)	SZC 10 g (*n* = 36)	Placebo (*n* = 33)	Total (*N* = 103)
Age, years, mean (SD)	72.4 (7.9)	71.1 (7.6)	76.1 (6.8)	73.2 (7.7)
Male, *n* (%)	26 (76.5)	28 (77.8)	23 (69.7)	77 (74.8)
Japanese race, *n* (%)	34 (100)	36 (100)	33 (100)	103 (100)
Weight, kg, mean (SD)	63.2 (11.6)	64.6 (14.7)	61.2 (10.7)	63.0 (12.5)
sK^+^ concentration, mmol/L, mean (SD)	5.6 (0.4)	5.5 (0.4)	5.7 (0.4)	5.6 (0.4)
sK^+^ concentration group, mmol/L, *n* (%)				
< 5.3	10 (29.4)	12 (33.3)	6 (18.2)	28 (27.2)
5.3–5.5	6 (17.6)	12 (33.3)	12 (36.4)	30 (29.1)
> 5.5	18 (52.9)	12 (33.3)	15 (45.5)	45 (43.7)
eGFR, mL/min/1.73 m^2^,^a^ mean (SD)	24.2 (14.3)	29.4 (16.6)	24.5 (13.5)	26.1 (14.9)
eGFR group, mL/min/1.73 m^2^,^a^ *n* (%)				
< 15	10 (29.4)	9 (25.0)	11 (33.3)	30 (29.1)
15– < 30	17 (50.0)	13 (36.1)	11 (33.3)	41 (39.8)
30– < 60	7 (20.6)	11 (30.6)	11 (33.3)	29 (28.2)
≥ 60	0 (0.0)	3 (8.3)	0 (0.0)	3 (2.9)
Serum bicarbonate, mmol/L, mean (SD)	19.9 (2.8)	20.3 (3.2)	19.4 (3.2)	NA^c^
Serum urea nitrogen, mmol/L, mean (SD)	14.8 (6.1)	12.8 (5.0)	14.6 (6.6)	NA^c^
Comorbidity,^b^ *n* (%)				
Chronic kidney disease	26 (76.5)	26 (72.2)	26 (78.8)	78 (75.7)
Diabetes	22 (64.7)	24 (66.7)	16 (48.5)	62 (60.2)
Heart failure	7 (20.6)	3 (8.3)	4 (12.1)	14 (13.6)
Concomitant medication use, *n* (%)				
RAASi therapy	27 (79.4)	26 (72.2)	27 (81.8)	80 (77.7)
Diuretics	17 (50.0)	9 (25.0)	11 (33.3)	37 (35.9)

*eGFR* estimated glomerular filtration rate, *NA* not available, *RAASi* renin–angiotensin–aldosterone system inhibitor, *SD* standard deviation *sK*^+^ serum potassium, *SMQ* Standardized Medical Dictionary for Regulatory Activities Queries, *SZC* sodium zirconium cyclosilicate

^a^eGFR estimated using the equation generated by the Japanese Society of Nephrology, with serum creatinine measured at the randomization visit

^b^Based on medical history by SMQ (narrow) search (ongoing)

^c^Not calculated

Most patients (97.1%) were compliant with the study medication; three patients in the placebo group did not meet treatment compliance parameters.

### Exponential rate of change in sK^+^ concentration at 48 h

At 48 h, exponential rates of change in sK^+^ concentration from baseline were reduced by 0.27% and 0.51% per hour with SZC 5 g and 10 g, respectively. These improvements were significantly greater with SZC 5 g (− 0.00261) and 10 g (− 0.00496) versus placebo (both *P* < 0.0001; Table 2). Numerical comparison of the slope values for SZC 5 g and 10 g suggested dose dependency.

**Table 2:** Exponential rate of change in sK^+^ concentration per hour from baseline to 48 h after administration of SZC (full analysis set)

Treatment group	Exponential rate of change			Comparison with placebo			*P* value
	Estimate	SE	95% CI	Estimate	SE	95% CI	
SZC 5 g (*n* = 34)	− 0.00273	0.000276	− 0.00328 to − 0.00219	− 0.00261	0.000385	− 0.00337 to − 0.00185	< 0.0001
SZC 10 g (*n* = 36)	− 0.00508	0.000269	− 0.00561 to − 0.00455	− 0.00496	0.000380	– 0.00571 to − 0.00420	< 0.0001
Placebo (*n* = 33)	− 0.00012	0.000288	− 0.00069 to 0.00045	–	–	–	–

Negative exponential rate of change relative to placebo indicates more rapid reduction (correction) of sK^+^. Serial log_e_ (sK^+^ concentration) values from 0 to 48 h were modeled using a random coefficient model, including fixed effects of intercept, time, time by treatment, and patient-level random effects for time and intercept

*CI* confidence interval, *SE* standard error, *sK*^+^ serum potassium, *SZC* sodium zirconium cyclosilicate

Results of a sensitivity analysis evaluating the effect of different potassium analytical assays on the primary endpoint were consistent with the primary analysis (Supplemental Table 1).

The exponential rate of change was greater in patients in both SZC groups versus placebo across patient subgroups (Supplemental Table 2).

### Proportions of patients with normokalemia

At 48 h, more patients were normokalemic in both the SZC 5 g (85.3%) and 10 g (91.7%) groups versus placebo (15.2%; Table 3). The odds ratios [OR; 95% confidence intervals (CI)] relative to placebo were 46.5 (10.1–213.2) and 71.8 (13.5–382.3), respectively (nominal *P* < 0.0001 for both). Results of the sensitivity analysis using i-STAT potassium data (Supplemental Table 3) as well as those from the subgroup analysis (Supplemental Fig. 1) were consistent with the overall results.

**Table 3:** Proportion of normokalemic patients at 48 and 24 h after administration of SZC (full analysis set)

Treatment group	Time point, h	Patients with normokalemia, *n* (%)	Comparison with placebo^a^		
			OR	95% CI	*P* value^b^
SZC 5 g (*n* = 34)	24	12 (35.3)	1.3	0.4–4.3	0.6167
	48	29 (85.3)	46.5	10.1–213.2	< 0.0001
SZC 10 g (*n* = 36)	24	30 (83.3)	15.3	4.0–58.7	< 0.0001
	48	33 (91.7)	71.8	13.5–382.3	< 0.0001
Placebo (*n* = 33)	24	9 (27.3)	–	–	–
	48	5 (15.2)	–	–	–

Patients with missing sK^+^ concentration data were regarded as not normokalemic

*CI* confidence interval, *OR* odds ratio, *sK*^+^ serum potassium, *SZC* sodium zirconium cyclosilicate

^a^Logistic regression model including treatment and baseline sK^+^ concentration as explanatory variables were used

^b^Nominal *P* value

The odds of achieving normokalemia at 24 h was greater for the SZC 10 g group versus placebo: OR 15.3 (95% CI 4.0–58.7); nominal *P* < 0.0001 (Table 3).

Following administration of SZC 10 g, 47.2% of patients attained normokalemia within 1 h, and a higher proportion of patients were normokalemic at all time points throughout the treatment period compared with placebo (Fig. 3). Following SZC 5 g, a higher proportion of patients were normokalemic from 4 h post-dose onwards compared with placebo. Similar results were achieved across patient subgroups (data not shown).

**Fig. 3:**
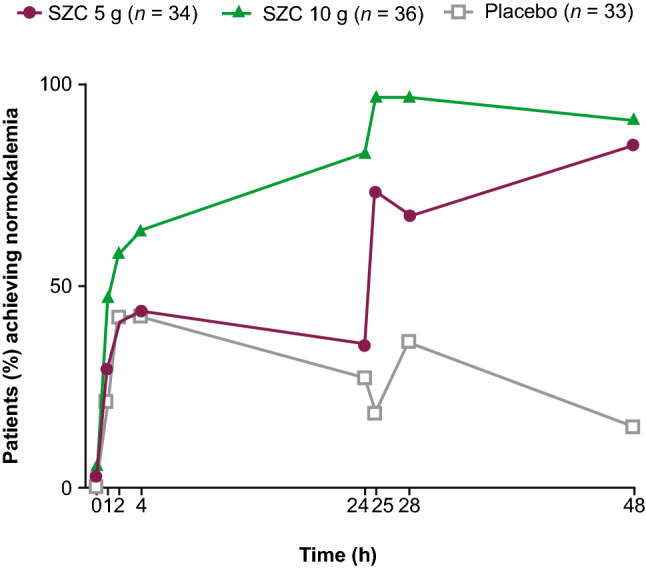
Proportion of normokalemic patients over time after administration of SZC (full analysis set). Normokalemia defined as sK^+^ 3.5–5.0 mmol/L. *sK*^+^ serum potassium, *SZC* sodium zirconium cyclosilicate

### Mean change from baseline in sK^+^ concentration

Mean sK^+^ concentration decreased from baseline to a greater degree with SZC 5 g and 10 g versus placebo over time (Fig. 4). Differences in the sK^+^-lowering effect were observed at 1 h after the first dose of SZC 10 g (mean change from baseline numerical reduction of − 0.37 mmol/L for SZC 10 g vs. − 0.13 mmol/L for placebo). During study day 2 (from 24 to 48 h), numerically greater mean reductions in sK^+^ concentration were observed at all time points in the SZC 10 g and 5 g dose groups than with placebo (Fig. 4). Reductions in sK^+^ from baseline were observed with SZC regardless of CKD stage, and results were consistent across patient subgroups (data not shown).

**Fig. 4:**
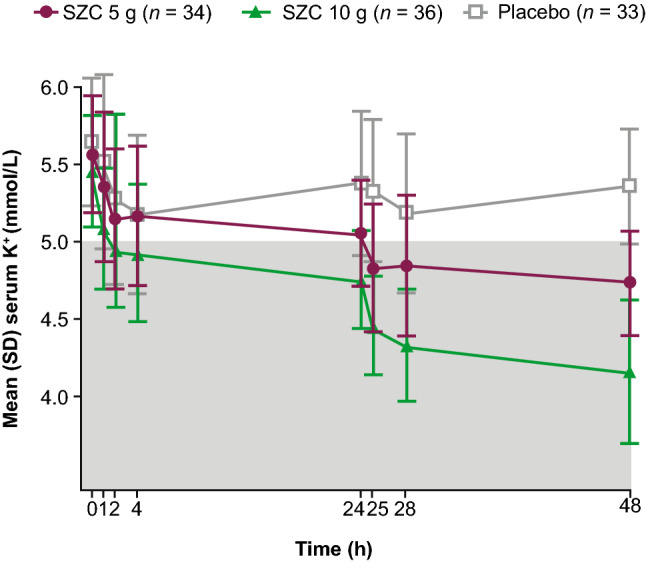
sK^+^ concentrations (mmol/L) over time after administration of SZC (mean ± SD) (full analysis set). The shaded area of the graph represents the range for normokalemia (sK^+^ 3.5–5.0 mmol/L). *SD* standard deviation, *sK*^+^ serum potassium, *SZC* sodium zirconium cyclosilicate

### Time to normalization of sK^+^ concentration

Median time to normalization of sK^+^ concentration was shorter with SZC 10 g versus placebo (1.8 h vs. 3.9 h; log-rank nominal *P* = 0.0006), and was similar for the SZC 5 g and placebo groups (3.9 h and 3.9 h, respectively; log-rank nominal *P* = 0.0586; Supplemental Fig. 2).

### Safety

Treatment-emergent AEs (TEAEs) were reported in four (11.8%), five (13.9%), and one (3.0%) patient in the SZC 5 g, SZC 10 g, and placebo groups, respectively (Table 4). All TEAEs were mild. Apart from one patient who experienced five episodes of tremor, AEs were single occurrences. TEAEs considered by the Investigator to be SZC-related were reported in two (5.6%) patients in the SZC 10 g group (tremor and ventricular extrasystole) and one (2.9%) patient in the SZC 5 g group (constipation). No TEAEs of hypokalemia, death, serious AEs, AEs leading to discontinuation, or other AEs deemed significant were reported.

**Table 4:** AEs (safety analysis set)

AEs, *n* (%)	SZC 5 g (*n* = 34)	SZC 10 g (*n* = 36)	Placebo (*n* = 33)
Any AE	4 (11.8)	5 (13.9)	1 (3.0)
Constipation	1 (2.9)	0 (0.0)	0 (0.0)
Erythema	0 (0.0)	1 (2.8)	0 (0.0)
Hypoglycemia	0 (0.0)	1 (2.8)	0 (0.0)
Nephrogenic anemia	1 (2.9)	0 (0.0)	0 (0.0)
Respiratory tract infection	0 (0.0)	0 (0.0)	1 (3.0)
Tension headache	0 (0.0)	1 (2.8)	0 (0.0)
Thirst	1 (2.9)	0 (0.0)	0 (0.0)
Tremor	0 (0.0)	1 (2.8)	0 (0.0)
Ventricular extrasystoles	0 (0.0)	1 (2.8)	0 (0.0)
Viral upper respiratory tract infection	1 (2.9)	0 (0.0)	0 (0.0)
SAEs	0 (0.0)	0 (0.0)	0 (0.0)
Death	0 (0.0)	0 (0.0)	0 (0.0)
Any AE leading to discontinuation of study drug	0 (0.0)	0 (0.0)	0 (0.0)
Any causally related AE	1 (2.9)	2 (5.6)	0 (0.0)

Patients with multiple events in the same category were counted only once in that category. Patients with events in more than one category were counted once in each of those categories. AEs with an onset date on or after first dose of study drug, but on or before day 3, are included as treatment-emergent AEs. Percentages are based on the total numbers of patients in the treatment group (*N*)

*AE* adverse event, *SAE* serious adverse event, *SZC* sodium zirconium cyclosilicate

No clinically relevant trends were apparent in the mean changes from baseline in hematology values, except for sK^+^. Dose-related increases in mean bicarbonate levels were observed with SZC 5 g (0.64 mmol/L) and SZC 10 g (1.49 mmol/L) compared with placebo (− 0.46 mmol/L) on study day 3. Two patients in the SZC 10 g group had a lowest sK^+^ concentration of < 3.5 mmol/L, based on central laboratory assessment, during day 1 to day 3; one of the two patients developed sK^+^ concentration < 3.0 mmol/L. Values returned to within the normal range by 6 days after dosing. No TEAEs were reported in either patient.

No clinically relevant dose-related changes from baseline in body weight, blood pressure, or heart rate were observed. An overall mean increase from baseline in QTc (corrected by Bazett's, QTcB) was seen on day 3 of 8.8 ms (SZC 5 g) and 15.6 ms (SZC 10 g), compared with a decrease of 0.6 ms with placebo. The proportions of patients who experienced a change in QTcB interval > 30 ms on study day 3 were: SZC 5 g, 5.9%; SZC 10 g, 16.7%; and placebo, 0.0%. No abnormal ECG findings were reported for either of the two patients who developed transient hypokalemia.

## Discussion

This is the first randomized controlled study to report the efficacy and safety of different doses of SZC in Japanese patients with hyperkalemia. SZC was effective in reducing sK^+^, supporting the use of SZC in this population. The efficacy endpoints evaluated were consistent with those from previous phase 3 studies of SZC [18, 20], enabling an indirect comparison of efficacy across different populations.

Our study met its primary efficacy endpoint; both SZC 5 g and 10 g resulted in significantly greater exponential decreases in sK^+^ concentration from baseline at 48 h versus placebo. The exponential rate of change was selected as a more clinically relevant endpoint than absolute change from baseline, since it incorporates the time to onset of effect and all sK^+^ measurements throughout the 48 h. The reductions in this study are comparable with those seen in previous phase 3 studies of SZC (i.e*.*, ~ 0.2–0.3% per hour) [18, 20].

The results of the secondary efficacy analyses support the primary analysis and indicate that SZC is effective for the rapid correction of sK^+^. Following administration of SZC 10 g, reductions in sK^+^ concentration were observed as early as 1 h after baseline, median time to normalization was < 2 h, and most patients were normokalemic at 24 h. Consistent with phase 3 studies of SZC in the non-Japanese population, almost all patients who received SZC 10 g (91.7%) were normokalemic at 48 h [18, 20]. Furthermore, our results suggest dose-dependent efficacy, with SZC 10 g being more efficacious in normalizing sK^+^ than 5 g, as observed previously [18, 20, 21]. These results were achieved without dietary restriction of potassium intake.

Treatment with placebo resulted in an unsustained reduction in mean sK^+^ concentration, with the lowest measurements in the placebo group observed 4 h post-administration. Acute reduction of sK^+^ within the first 4 h of placebo administration has been observed in an earlier study of SZC [18]. The mechanism of this reduction is unknown, and could possibly reflect the circadian rhythm of sK^+^ concentration [28] or abrupt changes in diet and daily routine due to the interventions within the clinical trial environment.

The overall safety and tolerability profile for SZC in the first 48 h of treatment was favorable, consistent with that observed in the non-Japanese population described previously; no new safety concerns were identified [18, 19]. Despite the sK^+^-lowering effects of SZC, no TEAEs of hypokalemia were reported. Two patients experienced transient sK^+^  < 3.5 mmol/L following treatment with 10 g SZC; however, normokalemia was re-established within 6 days. No patients reported TEAEs of edema, which have been observed in previous clinical trials [23, 24]. The increase in QTc interval observed in patients treated with SZC was considered clinically insignificant and was consistent with changes reported in a previous phase 3 study of SZC [18]. QT interval is known to be shortened in hyperkalemia and to lengthen with the decreasing sK^+^ concentration as hyperkalemia is corrected [29]. Overall, the efficacy and tolerability data for SZC in the Japanese population suggest that acute SZC treatment for 48 h, at the doses recommended, is a valid treatment option for hyperkalemia in the Japanese population.

Hyperkalemia is common in patients receiving RAASi [3] and can prevent at-risk patients (e.g., those with HF or CKD) from receiving the optimal dose of RAASi required to improve survival. SZC normalized sK^+^ in patients receiving RAASi, suggesting that SZC may avoid the need for dose reduction or discontinuation of RAASi in patients experiencing hyperkalemia.

Patients with CKD, HF, and diabetes are prone to hyperkalemia [2, 3, 6, 7]. As such, our study included patients with many concomitant diseases and treatments; proportions of patients at baseline with comorbid CKD, diabetes, or receiving RAASi were high, reflecting their real-world prevalence among Japanese patients with hyperkalemia [1]. This is also consistent with the study groups included in phase 3 studies of SZC in the non-Japanese population [18, 20, 21]. Fewer patients in our study had HF at baseline compared with other studies of SZC [18, 20], potentially reflecting the lower rate of HF typically seen in Japanese patients compared with patients from other countries [30]. Our study can, therefore, be considered representative of the general Japanese population of patients with hyperkalemia.

Limitations include, first, that the study was conducted among ambulatory patients, and patients who had potassium concentrations > 6.5 mmol/L or required dialysis were excluded. Therefore, the efficacy of SZC in these groups and in hospitalized patients among the Japanese population could not be determined. Second, clinical endpoints other than sK^+^ concentration were not assessed. Finally, the efficacy of maintenance therapy with SZC was not assessed. However, the efficacy of SZC in the dialysis [31] and emergency settings [32], as well as the impact of SZC on RAASi dosing [22], in predominantly non-Asian populations has been reported previously. Furthermore, the short-term study duration reflects the urgency with which hyperkalemia needs to be corrected and the rapidity with which SZC exerts its effects. Further studies have been conducted to evaluate the long-term efficacy and tolerability of SZC as well as the optimal SZC dose required for maintenance therapy in the Japanese population (NCT03172702).

In conclusion, SZC at both 5 g and 10 g given TID for 48 h was efficacious in reducing sK^+^ in Japanese patients with hyperkalemia and provided rapid attainment of normokalemia. SZC was well tolerated and no new safety concerns were identified. The results support the approval of SZC for the management of hyperkalemia in Japanese patients.

## Electronic supplementary material

Below is the link to the electronic supplementary material.

Supplementary file1 (DOCX 318 kb)
