# Supplementary material for: Correction of serum potassium with sodium zirconium cyclosilicate in Japanese patients with hyperkalemia: a randomized, dose–response, phase 2/3 study
Source: Clin Exp Nephrol. 2020 Aug 10;24(12):1144–53. doi: 10.1007/s10157-020-01937-1 (PMC7599176; doi:10.1007/s10157-020-01937-1)
Supplement: Supplementary file 1 — Supplementary file1 (DOCX 318 kb) [file 10157_2020_1937_MOESM1_ESM.docx]

**Correction of serum potassium with sodium zirconium cyclosilicate in Japanese patients with hyperkalemia: A randomized, dose-response, phase 2/3 study**

Naoki Kashihara,^1^ Toshiki Nishio,^2^ Takeshi Osonoi,^3^ Yosuke Saka,^4^ Toshiyuki Imasawa,^5^ Takayasu Ohtake,^6^ Hiroshi Mizuno,^7^ Yugo Shibagaki,^8^ Hyosung Kim,^9^ Toshitaka Yajima,^9^ Nobuaki Sarai^9^

^1^Department of Nephrology and Hypertension, Kawasaki Medical School, Okayama, Japan; ^2^Nephrology Dialysis Center, Kusatsu General Hospital, Shiga, Japan; ^3^Department of Internal Medicine, Nakakinen Clinic, Ibaraki, Japan; ^4^Department of Nephrology, Kasugai Municipal Hospital, Aichi, Japan; ^5^Department of Nephrology, Chiba-Higashi Hospital, Chiba, Japan; ^6^Department of Nephrology, Shonan Kamakura General Hospital, Kanagawa, Japan; ^7^Department of Nephrology, Inage Hospital, Chiba, Japan; ^8^Division of Nephrology and Hypertension, St. Marianna University School of Medicine Hospital, Kanagawa, Japan; ^9^Research and Development, AstraZeneca K.K., Osaka, Japan

# Supplemental Material

**Table of Contents**

[Supplemental Material 2](#_Toc37164863)

[Methods 5](#_Toc37164864)

[Randomization and blinding 5](#_Toc37164865)

[Ethical permissions and patient consent 5](#_Toc37164866)

[Patients 6](#_Toc37164867)

[Inclusion criteria 6](#_Toc37164868)

[Exclusion criteria 6](#_Toc37164869)

[Study assessments 8](#_Toc37164870)

[Screening 8](#_Toc37164871)

[Potassium samples 8](#_Toc37164872)

[Laboratory safety measures 9](#_Toc37164873)

[Physical examination 9](#_Toc37164874)

[ECG 10](#_Toc37164875)

[Vital signs 10](#_Toc37164876)

[Concomitant medication 10](#_Toc37164877)

[Study endpoints 11](#_Toc37164878)

[Baseline sK^+^ measurement 11](#_Toc37164879)

[Adverse events 11](#_Toc37164880)

[Compliance 11](#_Toc37164881)

[Statistical analysis 12](#_Toc37164882)

[Sample size calculation 12](#_Toc37164883)

[Missingness of sK^+^ data 12](#_Toc37164884)

[Primary efficacy endpoint 12](#_Toc37164885)

[Key secondary efficacy endpoint 14](#_Toc37164886)

[Other secondary efficacy endpoints 14](#_Toc37164887)

[Sensitivity analyses 14](#_Toc37164888)

[Subgroup analyses 14](#_Toc37164889)

[Safety 15](#_Toc37164890)

[Results 15](#_Toc37164891)

[Treatment exposure 15](#_Toc37164892)

[Supplemental Table 1. Sensitivity analysis using i-STAT potassium data: Exponential rate of change in potassium concentration from baseline to 48 hours after administration of SZC (full analysis set) 16](#_Toc37164893)

[Supplemental Table 2. Subgroup analysis: Exponential rate of change in sK^+^ concentration from baseline to 48 hours after administration of SZC (full analysis set) 17](#_Toc37164894)

[Supplemental Table 3. Sensitivity analysis using i-STAT potassium data: Proportion of normokalemic patients at 48 hours after administration of SZC (full analysis set) 20](#_Toc37164895)

[Supplemental Figure 1. Subgroup analysis: Proportion of normokalemic patients at 48 hours after administration of SZC (full analysis set). 21](#_Toc37164896)

[Supplemental Figure 2. Time to normalization of sK^+^ (3.5–5.0 mmol/L), Kaplan–Meier plot (full analysis set). 22](#_Toc37164897)

[References 23](#_Toc37164898)

# Methods

## Randomization and blinding

Randomization was performed using randomization codes and an interactive voice response system/interactive web response system. Randomization codes were generated in blocks of six to ensure balance (1:1:1) between the three treatment arms. Randomization was stratified according to kidney function (estimated glomerular filtration rate [eGFR] < 60 mL/min/1.73 m^2^ or ≥60 mL/min/1.73 m^2^), measured at each study center during the screening period, so that the proportion of patients with eGFR ≥ 60 mL/min/1.73 m^2^ did not exceed 25% of the entire randomized population.

Study site staff and patients were blinded to treatment assignment. Study treatments were provided as a powder for oral suspension in a sachet. The exterior appearance of the sachets was identical, but the volume of study drug differed depending upon the randomized treatment group. Individual sachets were enclosed in a carton with a tamper evident seal intended to be broken exclusively by patients just before taking the study drug.

### Ethical permissions and patient consent

The study was performed in accordance with the Declaration of Helsinki, International Council for Harmonisation, and Good Clinical Practice. The informed consent form, protocol, and amendments were approved by an institutional review board and national regulatory authority prior to study initiation. All participants provided written informed consent.

## Patients

Patients who consented to participate in the study were assessed during the screening visit to ensure that the study eligibility criteria were met.

### Inclusion criteria

1. Provision of informed consent prior to any study-specific procedures
2. Patients aged ≥ 18 years. For patients aged < 20 years, written informed consent had to be obtained from the patient and his or her legally acceptable representative
3. Two consecutive i-STAT potassium values measured 60 (± 10) minutes apart, both values had to be ≥ 5.1 mmol/L and ≤ 6.5 mmol/L, and measured within 1 day before the first dose of study drug on study day 1
4. Ability to have repeated blood draws or effective venous catheterization
5. To prevent pregnancy, female patients had to be 1-year postmenopausal, surgically sterile, or using an acceptable method of contraception (defined as a barrier method in conjunction with a spermicide) for the duration of the study (from the time they signed consent) and for 3 months after the last dose of sodium zirconium cyclosilicate (SZC)/placebo. In addition, oral contraceptives, approved contraceptive implant, long-term injectable contraception, intrauterine device, or tubal ligation were allowed. Oral contraception alone was not acceptable; additional barrier methods in conjunction with spermicide had to be used

### Exclusion criteria

1. Involvement in the planning and/or conduct of the study
2. Cause or symptoms of pseudohyperkalemia, such as:

- Hemolyzed blood specimen due to excessive fist clenching to make veins prominent
- Hemolyzed blood specimen due to difficult or traumatic venepuncture
- History of severe leukocytosis or thrombocytosis

1. Patients treated with lactulose, rifaximin, or other non-absorbed antibiotics for hyperammonemia within 7 days prior to first dose of study drug on study day 1
2. Patients treated with resins (such as sevelamer hydrochloride, sodium polystyrene sulfonate, or calcium polystyrene sulfonate), calcium acetate, calcium carbonate, or lanthanum carbonate, within 7 days prior to the first dose of study drug
3. Patients with a life expectancy of < 3 months
4. Patients who were severely physically or mentally incapacitated and who, in the opinion of the Investigator, were unable to perform the tasks associated with the protocol
5. Female patients who were pregnant, lactating, or planning to become pregnant
6. Patients who had active or history of diabetic ketoacidosis
7. Presence of any condition which, in the opinion of the Investigator, placed the patient at undue risk or potentially jeopardized the quality of the data to be generated
8. Known hypersensitivity or previous anaphylaxis to SZC or to components thereof
9. Treatment with a drug or device within the last 30 days that had not received regulatory approval at the time of study entry
10. Patients with cardiac arrhythmias that required immediate treatment
11. Patients on dialysis

## Study assessments

### Screening

S-creatinine (local laboratory) was measured for stratification (based on eGFR value) prior to randomization. If eGFR assessments were performed within 1 month prior to visit 1, the latest one could be used for visit 1 data. The glomerular filtration rate was estimated using the following equation generated by the Japanese Society of Nephrology [1].

Glomerular filtration rate (mL/min/1.73 m^2^) = 194 × serum creatinine^−1.094^ × Age^−0.287^ (× 0.739 if female)

### Potassium samples

Patients fasted for a minimum of 8 hours prior to blood sampling, receiving nil by mouth except for water, coffee or tea, with or without milk and/or sugar, and essential medications.

Potassium samples were taken at the following intervals:

1. Day –1 to 1: first assessment to confirm qualification (within 1 day of the first administration of study drug); and 1-hour post first assessment
2. Day 1: 0 hours (pre-dose); 1-, 2-, and 4-hours post-dose 1; and 90 minutes post-dose 2
3. Day 2: 0 hours (pre-dose); 1- and 4-hours post-dose 1
4. Day 3: 0 hour
5. End of study: 0 hour

### Laboratory safety measures

Blood and urine samples for determination of clinical chemistry, hematology, and urinalysis were collected at visits 2, 4, and end of study. Serum clinical chemistry and hematology were measured fasting and before administration of study drug. Analysis was performed at the central laboratory. Laboratory safety variables included:

1. Blood: hemoglobin, hematocrit, erythocyte count, total leukocyte count, and platelet count
2. Serum: total protein, albumin, bicarbonate, blood urea nitrogen, creatinine, bilirubin, alkaline phosphatise, glucose, sodium, potassium, inorganic phosphate, calcium, magnesium, gamma-glutamyl transferase, aspartate aminotransferase, and alanine aminotransferase
3. Urine: pH, specific gravity, glucose, ketones, bilirubin, urobilinogen, blood, albumin, creatinine, and human chorionic gonadotropin (only for females of childbearing potential)

### Physical examination

A complete physical examination was performed no earlier than 1 day before administration of the first dose of study drug on visit 2 and end of study visit. The complete physical examination included the following: general appearance, including skin, height, and weight; lymph nodes; thyroid; musculoskeletal/extremities; cardiovascular examinations, including assessment of signs of heart failure; lungs; abdomen; and neurological systems.

### ECG

A 12-lead ECG was performed after the patient had been lying down for 5 minutes. Heart rate, P and QRS durations, PR and QT intervals were recorded from the standard lead of the computerized quantitative 12-lead ECG. ECGs were recorded at visits 2, 4, and end of study. In addition, for patients who had i-STAT potassium concentrations ≥6.1 mmol/L at the 4-hour post‑first dose time point on day 1 (visit 2), an additional ECG was recorded 90 minutes post-second dose.

### Vital signs

Pulse rate and systolic and diastolic blood pressure (BP) were assessed using non‑invasive equipment by an adequately trained healthcare professional. The disappearance of sound (Korotkov phase V) was used for the diastolic reading. Three readings separated by 2 minutes were averaged. If the first two readings of systolic BP differed by > 5 mmHg, additional readings were obtained. BP was checked in both arms at the first visit. Subsequent BP measurements were recorded in the arm with the higher pressure. BP was measured in either supine or sitting position, which was adopted for at least 5 minutes.

## Concomitant medication

Drugs that were required to be taken 2 hours before or after study drug to avoid a possible raised gastric pH drug interaction are listed below:

1. Azole antifungals: ketoconazole, itraconazole, posaconazole, and voriconazole
2. Anti-HIV drugs: atazanavir, nelfinavir, indinavir, ritonavir, saquinavir, raltegravir, ledipasvir, and rilpivirine
3. Tyrosine kinase inhibitors: erlotinib, dasatinib, and nilotinib

## Study endpoints

### Baseline sK^+^ measurement

The baseline measurement for sK^+^ used for the efficacy analyses was established by taking the mean of two different sK^+^ concentrations, recorded 60 minutes apart (to confirm qualification for randomization) and then averaged with the last sK^+^ concentration taken just before administration of the first dose (0 hours) on day 1.

### Adverse events

All adverse events (AEs) were classified using the Medical Dictionary for Regulatory Activities version 20.0. AEs with an onset date on or after first dose of study drug, but on or before day 3, were considered as treatment-emergent AEs. Variables collected for AEs included:

1. whether the AE was serious or non-serious
2. maximum intensity
3. outcome (death or no death)
4. whether the AE led to treatment discontinuation or not
5. investigator causality rating

### Compliance

Treatment compliance was defined as the number of sachets actually taken divided by the number of sachets that should have been taken. A patient was considered compliant if percent compliance was between ≥ 80% and ≤ 120%.

## Statistical analysis

### Sample size calculation

The target sample size for the primary efficacy endpoint was calculated based on a random slope model, with parameter estimates based on a previous phase 3 SZC study.[2] Based on these parameters, 102 patients (34 patients per group) provided more than 95% power to detect the difference in slopes of 0.055/day (assumed for SZC 10 g vs. placebo) and 83% power to detect the difference in slopes of 0.030 per day (assumed for SZC 5 g vs. placebo).

### Missingness of sK^+^ data

Missing central laboratory sK^+^ data were replaced with point-of-care i‑STAT potassium data by adjusting for the mean paired difference between the centrally obtained and i-STAT values collected at the same time point. If both central laboratory and i-STAT data were missing, the measurement at that time point was deemed missing.

### Primary efficacy endpoint

The objective of the primary efficacy endpoint (exponential rate of change through to 48 hours) was to demonstrate superiority of SZC 10 g and 5 g compared with placebo. The exponential rate of change referred to the slope coefficient when longitudinal serum potassium (sK^+^) concentrations on a log scale were regressed vs. time. Exponential rate of change in sK^+^ through to 48 or 24 hours was analyzed with a random coefficient model, using the SAS procedure PROC MIXED:

*Ln(Y_ijk_) = (α + a_ij_) + (β_0_ + β_1_L_i_ + β_2_H_i_ + b_ij_) ⋅ t_k_ + ε_ijk_*

Where:

1. *Y_ijk_* is the k_th_ measurement (at time *t_k_*) of patient *j* in treatment group *i*
2. *α* is the fixed intercept
3. *a_ij_* is the random intercept for patient *j* in treatment group *i*
4. *β_0_* is the fixed slope on time *t* for reference group (placebo)
5. *β_1_* is the fixed slope difference of SZC 5 g three times daily (TID) to reference group (placebo)
6. *β_2_* is the fixed slope difference of SZC 10 g TID to reference group (placebo)
7. *L_i_* = 1 if the patient is in SZC 5 g TID and *L_i_* = 0 otherwise
8. *H_i_* = 1 if the patient is in SZC 10 g TID and *H_i_* = 0 otherwise
9. *b_ij_* is the random slope on time *t* for patient *j* in treatment *i*
10. Discrete time *t_k_* spans as follows, and was regarded as a continuous variable in the models:

- from 0 to 48 hours (0, 1, 2, 4, 5.5, 24, 25, 28, and 48 hours) in the primary analysis model
- from 0 to 24 hours (0, 1, 2, 4, 5.5, and 24 hours) in the secondary analysis model

For the purposes of these models, patient-level random effects were assumed to be bivariate normally distributed with mean zeros, independent of random error.

### Key secondary efficacy endpoint

The proportions of patients who achieved normokalemia at 24 and 48 hours were analyzed using a logistic regression model, with treatment as a factor and baseline sK^+^ concentration as a covariate. Findings are presented as odds ratio with 95% confidence intervals with nominal two-sided *p* values for each pairwise comparison. Patients with missing sK^+^ at that time point were counted as non-responders (i.e., not normokalemic).

### Other secondary efficacy endpoints

The mean change from baseline in sK^+^ concentration at all measured time intervals post-dose was summarized descriptively.

Median time to normalization of sK^+^ concentration (3.5–5.0 mmol/L) was estimated using Kaplan–Meier plots for the cumulative percentage of patients who achieved normokalemia. Patients who did not achieve normokalemia until 48 hours were censored at the time point when the last available sK^+^ concentration was obtained. Treatment comparisons were performed using a log-rank test with nominal two-sided *P* values.

### Sensitivity analyses

A sensitivity analysis was conducted for the primary efficacy and key secondary efficacy endpoints whereby i-STAT potassium values were used instead of central laboratory sK^+^ data to determine the effect of the different methods of sampling.

### Subgroup analyses

Subgroup analyses were also performed for the primary and secondary efficacy endpoints. Analyses for each endpoint were repeated for the following subgroups based on medical history: patients with or without HF, diabetes mellitus, or CKD; and patients treated or not treated with RAASi. An analysis of patients by CKD stage was also investigated for the secondary endpoint: Mean change and mean percent change from baseline in sK^+^.

### Safety

AE reporting was summarized by descriptive statistics.

For continuous variables (such as change from baseline over time in laboratory parameters), observed values and changes from baseline to day 3 and day 9 (end of study) were summarized by descriptive statistics. Categorical variables were summarized using the number (%) of patients in each category.

The incidence of hypokalemia was evaluated by summarizing the proportion of patients with a lowest sK^+^ concentration (regardless of scheduled or unscheduled, during day 1 to day 3 and during day 4 to end of study) of < 3.5 mmol/L and was repeated for a more severe definition of < 3.0 mmol/L.

# Results

## Treatment exposure

The median duration of exposure was 2 days in each of the SZC 5 g, SZC 10 g, and placebo groups. The median overall number of doses was six doses in each of the three treatment groups.

Supplemental Table 1. Sensitivity analysis using i-STAT potassium data: Exponential rate of change in potassium concentration from baseline to 48 hours after administration of SZC (full analysis set)

| Treatment group | Exponential rate of change | | | Comparison with placebo | | | *P* value |
| --- | --- | --- | --- | --- | --- | --- | --- |
|  | Estimate | SE | 95% CI | Estimate | SE | 95% CI |  |
| SZC 5 g (*n* = 34) | –0.00282 | 0.000277 | –0.00337 to –0.00227 | –0.00261 | 0.000378 | –0.00336 to –0.00186 | <0.0001 |
| SZC 10 g (*n* = 36) | –0.00526 | 0.000270 | –0.00580 to –0.00472 | –0.00506 | 0.000373 | –0.00580 to –0.00432 | <0.0001 |
| Placebo (*n* = 33) | –0.00020 | 0.000289 | –0.00078 to 0.00037 | – | – | – | – |

Negative exponential rate of change relative to placebo indicates more rapid reduction (correction) of i-STAT potassium.
Serial log_e_ (i-STAT potassium concentration) values from 0 to 48 hours were modeled using a random coefficient model including fixed effects of intercept, time, time by treatment, and patient-level random effects for time and intercept.
CI, confidence interval; SE, standard error; SZC, sodium zirconium cyclosilicate.

Supplemental Table 2. Subgroup analysis: Exponential rate of change in sK^+^ concentration from baseline to 48 hours after administration of SZC (full analysis set)

| Subgroup | Treatment group | Exponential rate of change | | | Comparison with placebo | | |
| --- | --- | --- | --- | --- | --- | --- | --- |
|  |  | Estimate | SE | 95% CI | Estimate | SE | 95% CI |
| **Heart failure** | | | | | | | |
| Yes | SZC 5 g (*n* = 7) | –0.00265 | 0.000745 | –0.00427 to –0.00102 | –0.00251 | 0.001239 | –0.00524 to 0.00022 |
|  | SZC 10 g (*n* = 3) | –0.00532 | 0.001140 | –0.00782 to –0.00283 | –0.00519 | 0.001510 | –0.00851 to –0.00186 |
|  | Placebo (*n* = 4) | –0.00014 | 0.000987 | –0.00229 to 0.00202 | – | – | – |
| No | SZC 5 g (*n* = 27) | –0.00277 | 0.000306 | –0.00338 to –0.00217 | –0.00266 | 0.000417 | –0.00349 to –0.00183 |
|  | SZC 10 g (*n* = 33) | –0.00506 | 0.000279 | –0.00561 to –0.00450 | –0.00495 | 0.000398 | –0.00574 to –0.00416 |
|  | Placebo (*n* = 29) | –0.00011 | 0.000306 | –0.00072 to 0.00049 | – | – | – |
| **Diabetes** | | | | | | | |
| Yes | SZC 5 g (*n* = 22) | –0.00256 | 0.000366 | –0.00329 to –0.00183 | –0.00260 | 0.000548 | –0.00369 to –0.00150 |
|  | SZC 10 g (*n* = 24) | –0.00511 | 0.000352 | –0.00582 to –0.00441 | –0.00515 | 0.000539 | –0.00623 to –0.00407 |
|  | Placebo (*n* = 16) | 0.00004 | 0.000437 | –0.00084 to 0.00091 | – | – | – |
| No | SZC 5 g (*n* = 12) | –0.00299 | 0.000433 | –0.00387 to –0.00211 | –0.00269 | 0.000564 | –0.00384 to –0.00155 |
|  | SZC 10 g (*n* = 12) | –0.00504 | 0.000433 | –0.00592 to –0.00417 | –0.00475 | 0.000564 | –0.00589 to –0.00361 |
|  | Placebo (*n* = 17) | –0.00029 | 0.000377 | –0.00106 to 0.00047 | – | – | – |
| **Chronic kidney disease** | | | | | | | |
| Yes | SZC 5 g (*n* = 26) | –0.00274 | 0.000287 | –0.00331 to –0.00217 | –0.00237 | 0.000402 | –0.00317 to –0.00157 |
|  | SZC 10 g (*n* = 26) | –0.00543 | 0.000287 | –0.00600 to –0.00486 | –0.00505 | 0.000402 | –0.00585 to –0.00425 |
|  | Placebo (*n* = 26) | –0.00037 | 0.000292 | –0.00096 to 0.00021 | – | – | – |
| No | SZC 5 g (*n* = 8) | –0.00289 | 0.000681 | –0.00429 to –0.00149 | –0.00352 | 0.000966 | –0.00553 to –0.00151 |
|  | SZC 10 g (*n* = 10) | –0.00400 | 0.000619 | –0.00527 to –0.00273 | –0.00463 | 0.000924 | –0.00655 to –0.00271 |
|  | Placebo (*n* = 7) | 0.00063 | 0.000773 | –0.00096 to 0.00222 | – | – | – |
| **RAASi use** | | | | | | | |
| Yes | SZC 5 g (*n* = 27) | –0.00297 | 0.000296 | –0.00356 to –0.00238 | –0.00279 | 0.000414 | –0.00362 to –0.00197 |
|  | SZC 10 g (*n* = 26) | –0.00503 | 0.000301 | –0.00563 to –0.00444 | –0.00486 | 0.000418 | –0.00569 to –0.00402 |
|  | Placebo (*n* = 27) | –0.00018 | 0.000307 | –0.00079 to 0.00043 | – | – | – |
| No | SZC 5 g (*n* = 7) | –0.00186 | 0.000723 | –0.00336 to –0.00037 | –0.00192 | 0.001032 | –0.00407 to 0.00024 |
|  | SZC 10 g (*n* = 10) | –0.00508 | 0.000612 | –0.00635 to –0.00381 | –0.00514 | 0.000958 | –0.00714 to –0.00314 |
|  | Placebo (*n* = 6) | 0.00006 | 0.000777 | –0.00155 to 0.00167 | – | – | – |

Negative exponential rate of change relative to placebo indicates more rapid reduction (correction) of sK^+^.
Serial log_e_ (sK^+^ concentration) values from 0 to 48 hours were modeled using a random coefficient model including fixed effects of intercept, time, time by treatment, and patient-level random effects for time and intercept.
CI, confidence interval; RAASi, renin-angiotensin-aldosterone system inhibitor; SE, standard error; sK^+^, serum potassium; SZC, sodium zirconium cyclosilicate.

Supplemental Table 3. Sensitivity analysis using i-STAT potassium data: Proportion of normokalemic patients at 48 hours after administration of SZC (full analysis set)

| Treatment group | Patients with normokalemia, *n* (%) | Comparison with placebo^a^ | | |
| --- | --- | --- | --- | --- |
|  |  | OR | 95% CI | *P* value^b^ |
| SZC 5 g (*n* = 34) | 30 (88.2) | 33.8 | 7.4 to 154.2 | <0.0001 |
| SZC 10 g (*n* = 36) | 34 (94.4) | 73.4 | 11.0 to 487.8 | <0.0001 |
| Placebo (*n* = 33) | 8 (24.2) | – | – | – |

^a^Logistic regression model including treatment and baseline i-STAT potassium concentration as explanatory variables were used.
^b^Nominal *P* value.
Patients with missing i-STAT potassium concentration data were regarded as not normokalemic at 48 hours.
CI, confidence interval; OR, odds ratio; SZC, sodium zirconium cyclosilicate.

Supplemental Figure 1. Subgroup analysis: Proportion of normokalemic patients at 48 hours after administration of SZC (full analysis set).


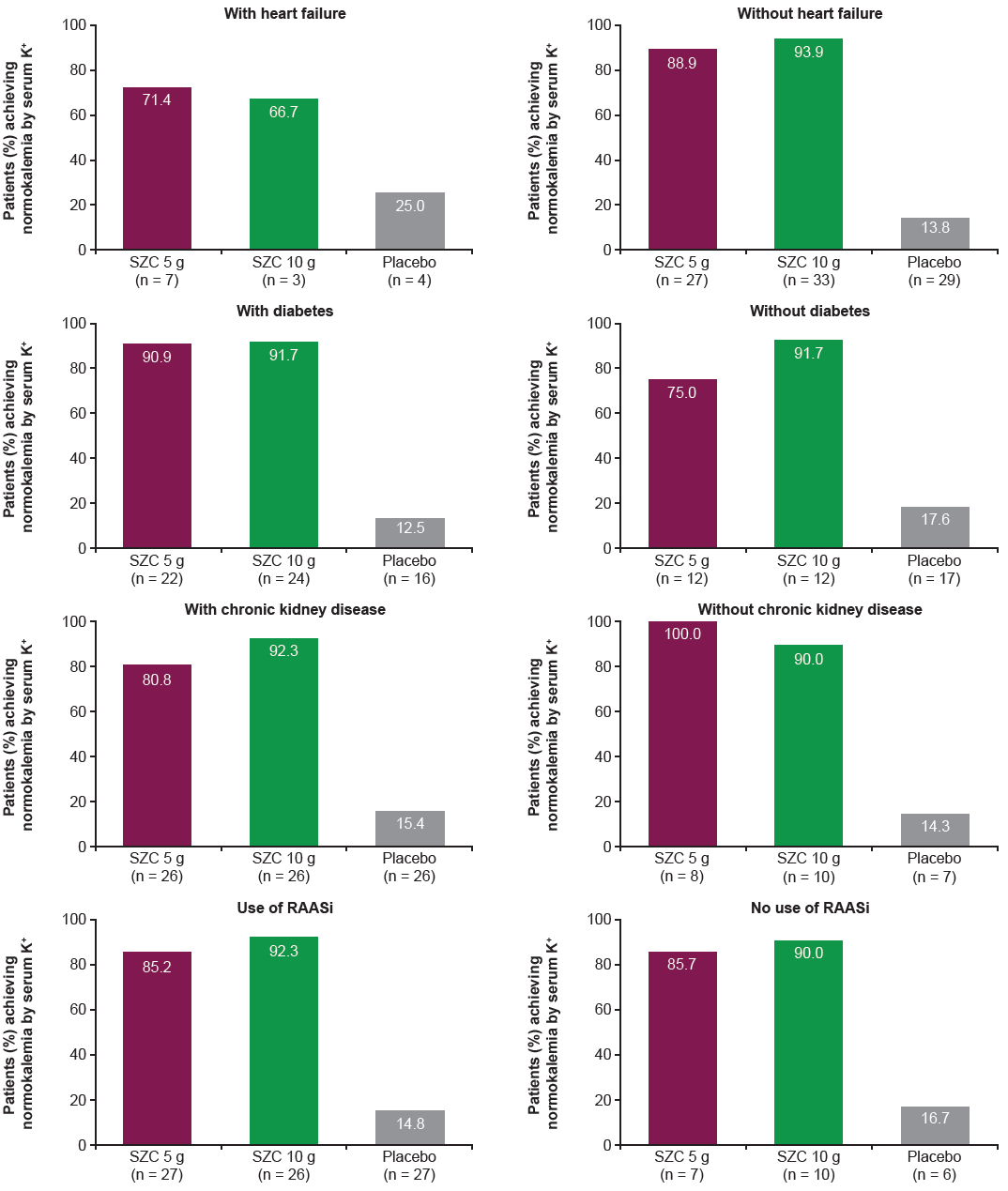


Normokalemia defined as sK^+^ 3.5–5.0 mmol/L.
Patients with missing sK^+^ concentration data were regarded as not normokalemic.
RAASi, renin-angiotensin-aldosterone system inhibitor; sK^+^, serum potassium; SZC, sodium zirconium cyclosilicate.

Supplemental Figure 2. Time to normalization of sK^+^ (3.5–5.0 mmol/L), Kaplan–Meier plot (full analysis set).


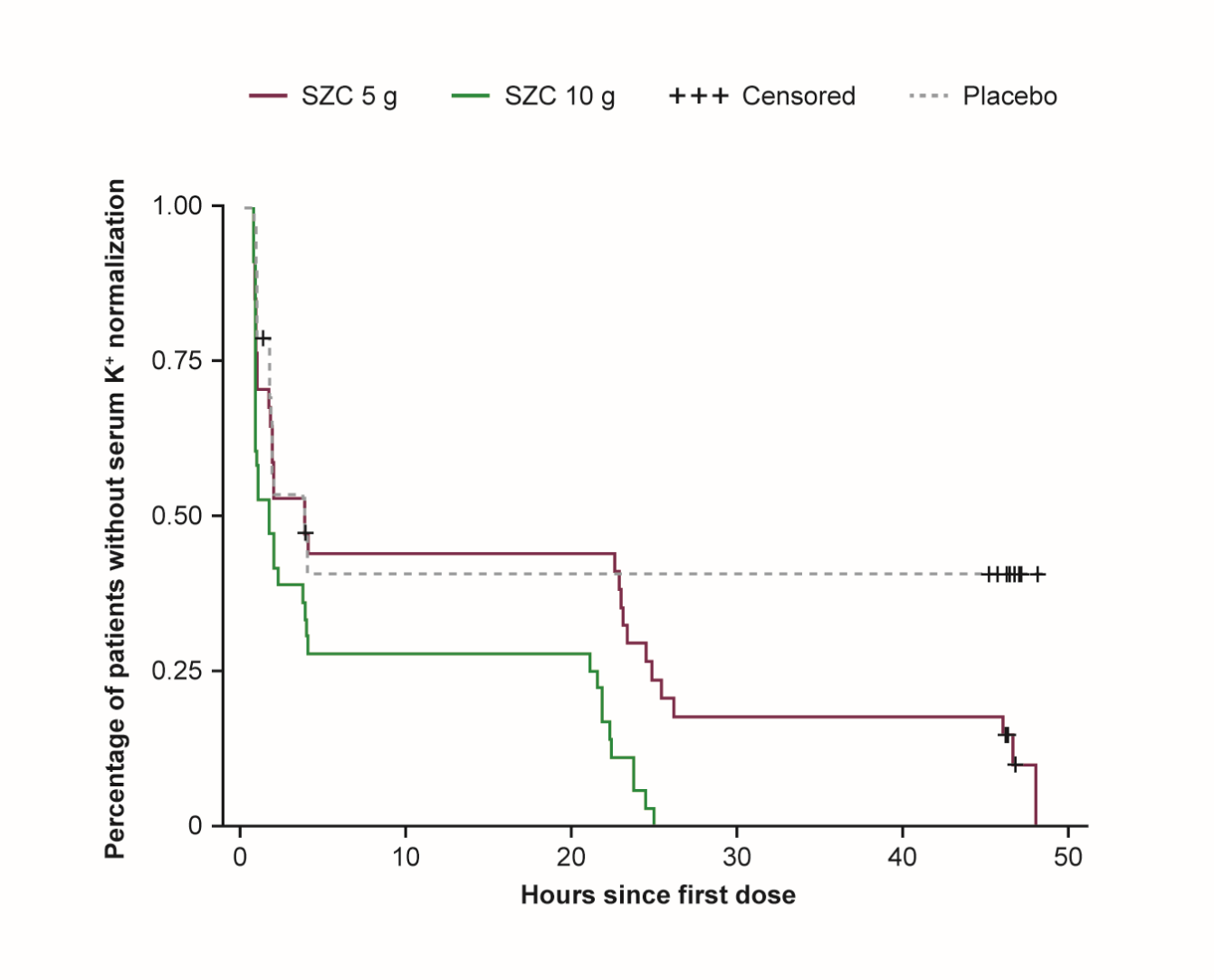


Patients who did not achieve normokalemia until hour 48 were censored at the time point when the last available sK^+^ measurement was obtained at or before hour 48.
sK^+^, serum potassium; SZC, sodium zirconium cyclosilicate.

# References

1. Matsuo S, Imai E, Horio M, Yasuda Y, Tomita K, Nitta K, et al. Revised equations for estimated GFR from serum creatinine in Japan. Am J Kidney Dis. 2009;53(6):982-92.

2. Packham DK, Rasmussen HS, Lavin PT, El-Shahawy MA, Roger SD, Block G, et al. Sodium zirconium cyclosilicate in hyperkalemia. N Engl J Med. 2015;372(3):222-31.
